# Major Polymorphisms of Genes Involved in Homocysteine Metabolism in Malaria Patients in Ouagadougou, Burkina Faso

**DOI:** 10.1155/2017/3468276

**Published:** 2017-05-18

**Authors:** Noé Yameogo, Bapio Valérie Elvira Jean Télesphore Bazie, Abdoul Karim Ouattara, Pouiré Yameogo, Tegwinde Rebeca Compaore, Dorcas Obiri-Yeboah, Florencia Wenkuuni Djigma, Simplice Damintoti Karou, Jacques Simpore

**Affiliations:** ^1^Laboratoire de Biologie Moléculaires et de Génétique (LABIOGENE), UFR-SVT, Université de Ouaga I Professeur Joseph KI-ZERBO, Ouagadougou, Burkina Faso; ^2^Département Substances Naturelles (DSN), Institut de Recherche en Sciences Appliquées et Technologies (IRSAT), Ouagadougou, Burkina Faso; ^3^Department of Microbiology and Immunology, School of Medical Sciences, University of Cape Coast, Cape Coast, Ghana; ^4^Ecole Supérieure des Techniques Biologiques et Alimentaires (ESTBA-UL), Université de Lomé, Lomé, Togo

## Abstract

This study analyzed the four main polymorphisms of the genes in homocysteine metabolism in malaria patients. Forty-two randomly selected subjects, diagnosed positive for* Plasmodium falciparum,* were included. The four genotypes were detected by real-time PCR using the MTHFR 677C>T, MTHFR 1298A>C, MTR 2756A>G, and MTRR 66A>G detection kit (Sacace Biotechnologies REF: T01002-96-S). The results revealed frequencies of 90% 677CC, 10% 677CT, and 00% 677TT for MTHFR C677T; 78.6% 1298AA, 19% 1298AC, and 2.4% 1298CC for MTHFR A1298C; 61.9% 2756AA, 33.3% 2756AG, and 4.8% 2756GG for MTR A2756G; and 50% of 66AA, 45% of 66AG, and 5% of 66GG for MTRR A66G. Correlations were found between A2756G MTR genotypes and parasitaemia (*P* = 0.02), MTRR A66G and hemoglobin genotypes (*P* = 0.009), and MTHFR A1298C and sex (*P* = 0.01). This study demonstrated for the first time an association between the A2756G MTR alleles and* Plasmodium falciparum *malaria in Burkina Faso and gave an overview of the genotypic distribution of the major SNPs influencing the metabolism of homocysteine.

## 1. Introduction

Homocysteine is an intermediate amino acid in the metabolism of methionine. The plasma level in homocysteine depends on many factors, including the folate, B12, and B6 vitamins status. In addition, the genetic polymorphisms of Methylene Tetrahydrofolate Reductase (MTHFR) play a crucial role. Hyperhomocysteinaemia is a risk factor for several pathologies such as cardiovascular disease [1, 2], neurodegenerative diseases [3], renal insufficiency, and non-insulin-dependent diabetes. Studies have shown that hyperhomocysteinaemia is more common in the Caucasian population with more than 15% of people with high homocysteinaemia [4]. The plasma concentration of homocysteine is lower in sub-Saharan African populations compared to the Mediterranean [5, 6]. This difference is due to the variable frequency of MTHFR polymorphisms between Westerners and Africans [7]. The frequency of the C677T allele varies with age in the African population. In Burkina Faso homocysteinaemia is low in the general population [5].

*Plasmodium falciparum*, the parasite responsible for the lethal cases of malaria, uses the pathway of polyamines essential to its proliferation and differentiation, thus imposing an oxidative stress on the host cell due to the use of glutathione [8, 9]. This therefore reduces the remethylation of homocysteine to methionine and promotes the transsulfuration pathway. This situation can however be influenced by the enzymes involved in the metabolism of homocysteine. Several enzymes are involved in methionine synthesis from homocysteine. These are methylenetetrahydrofolate reductase (MTHFR), methionine synthase (MTR), and methionine synthase reductase (MTRR).

Depending on the polymorphisms of the genes responsible for the synthesis of these enzymes, the route of the remethylation can be promoted over that of the transsulfuration and vice versa. The development of* Plasmodium* in human is therefore influenced by the polymorphisms of these genes which intervene in the remethylation of homocysteine into methionine. Indeed, when the route of remethylation is favored, homocysteinaemia is low causing a low production of glutathione and thus reducing the development of* Plasmodium* in the human body. On the other hand, if this pathway is blocked, hyperhomocysteinaemia leads to an increase in the transsulfuration pathway and an accumulation of glutathione in the organism necessary for* Plasmodium* development.

Studies on the possible association of the polymorphisms of genes involved in homocysteine metabolism and malaria in Burkina Faso are missing. However, Chillemi et al. (2005) suggested that the determination of plasma homocysteine levels could be used as a measure of the severity of malaria infection [7]. The genes involved in the metabolism of homocysteine are highly polymorphic but only four major SNPs significantly influence it. It is therefore necessary to study the effect of these SNPs on the malaria evolution in Burkina Faso. In this study, the polymorphisms of the MTHFR genes (MTHFR C677T and MTHFR A1298C), MTR (MTR A2756G), and MTRR (MTRR A66G) were determined in malaria patients in Burkina Faso.

## 2. Material and Methods

### 2.1. Study Area

The present study was a prospective study conducted from September to November 2014 in Koubri. This is a rural municipality located in the southern Ouagadougou at approximately 25 km, with the following geographical coordinates: 12°10′ north and 1°24′ west. Koubri has an average annual temperature of 28.2°C and it falls on average 780 mm of rain per year. However, the locality has numerous reservoirs of water that make the presence of mosquitoes vectors persistent throughout the year, so explaining the endemicity of malaria in the area.

### 2.2. Study Population

The study initially included 182 patients (1 to 79 years) who underwent malaria rapid diagnosis test, SD BIOLINE Malaria Ag P.f/Pan, which enables the detection of the* Plasmodium falciparum* HPRII antigen (histidine II-rich protein) and* Plasmodium* lactic dehydrogenase (pLDH) common to* Plasmodium* species in human whole blood. Tick smears were made for each patient for the confirmation of plasmodial species and the parasite count. Finally, the 42 patients with the highest parasite density (density superior to 3000 parasite/*μ*L of blood) were selected for the G-6-PD deficiency study. The age and the antimalarial drugs intakes were directly recorded prior to the blood sampling.

### 2.3. Blood Sampling

From the 42 selected patients, 5 mL of venous blood was collected in two EDTA impregnated tubes. The first tube was used for the hematological analysis; the second was centrifuged to discard plasma and the pellets were stored at −80°C until use for nucleic acid extractions.

### 2.4. Hematological Analysis

Platelet count and hemoglobin were determined with total venous blood using the ABX micro 60 automate. Hemoglobin electrophoresis was performed at alkaline pH (tris-glycine buffer pH 9.5) on cellulose acetate strip (CELLOGEL 5.7 × 14 cm). In brief, whole blood was washed three times with 0.9% NaCl by successive centrifugations at 3000 rpm for 5 min. Finally, the red cell pellet was lysed with 500 *μ*L of 1% saponin and the sample spots were deposited on the CELLOGEL with capillary tubes. The migration was made with 150 V in 60 min.

### 2.5. DNA Extraction and Genotyping of Polymorphisms

The extraction of DNA from the blood pellet was carried out using the salting out method [15, 16]. The Biodrop *μ*LITE (Isogen Life Science N.V./S.A, Temse, Belgium) was used for DNA extracts purity and final concentration assessment.

The samples genotyping with real time PCR was carried out using “Real Time PCR kit for detection of MTHFR 677C>T, MTHFR 1298A>C, MTR 2756A>G, and MTRR 66A>G” (Sacace Biotechnologies, REF: T01002-96-S). SaCycler-96 (Sacace Biotechnologies®; Como, Italy) was used for amplification. Thermocycling was performed at 80°C for 2 min and 94°C for 3 min and then 40 cycles at 94°C for 15 sec and 64°C (annealing temperature) for 40 sec.

### 2.6. Statistical Analysis

The data was processed using Excel 2010 (Microsoft) and SPSS® software version 20 (SPSS Inc., Chicago, USA). The Pearson Chi test was used for comparisons and any *P* value < 0.05 was considered statistically significant.

### 2.7. Ethical Considerations

The study was approved by the Ethics Committee for Health Research in Burkina Faso (Deliberation number 2014-9-128). Adults have given their free and informed consent for their participation in the study while the parents or guardians of the minors have given their approval for the participation of the minor.

## 3. Results

### 3.1. Sociodemographic and Paraclinical Data

The study involved 69.05% (29/42) men and 30.95% (13/42) women, the majority of whom were children with 14.28% (6/42) over 20 years of age (Table 1). The mean age was 14.22 ± 12.26 years. In the study population 52.38% (22/42) of the patients were on antimalarial treatment before the thick smear was performed, while 47.62% (20/42) of them did not yet receive any treatment. Among the screened patients, 54.76% presented a parasitaemia above 10,000 trophozoites/*μ*L while 11.90% had a parasitaemia between 3,000 and 10,000 trophozoite/*μ*L. However, the parasitaemia was below 3,000 trophozoites/*μ*L for 33.34% patients.

With regard to the anemia, 40.48% patients were not anemic (Hb > 12 g L^−1^), while 35.72% (15/42) had moderate anemia (10 < Hb < 12 g L^−1^) and 23.80% (10/42) had anemia (Hb < 10 g L^−1^). It should also be noted that 33.33% (14/32) of patients had thrombocytopenia compared to 66.67% (28/42) with normal platelet count. The frequency of the HbAA genotype was 57.14% (27/42) with one of the patients (0.02%) presenting major sickle cell syndrome (SC).

### 3.2. Gene Polymorphisms

**Figure 1:**
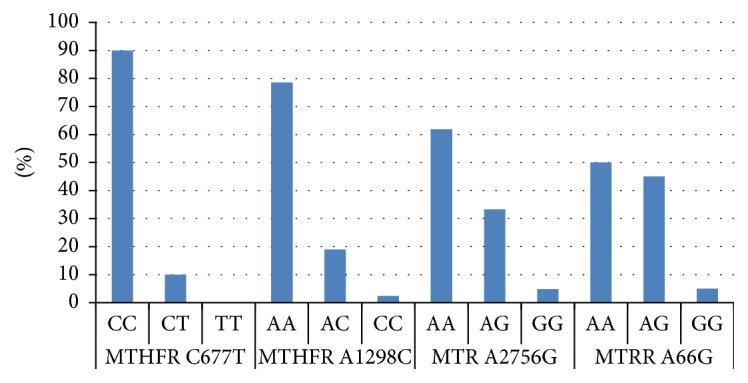
Polymorphisms distribution among screened people.

Figure 1 shows the PCR analysis of the four polymorphisms studied. The MTHFR A1298C, MTR A2756G, and MTRR A66G genes all showed homozygous mutants with the exception of MTHFR C677T. The correlation between different polymorphisms studied and hemoglobin genotypes gave a statistically significant difference for the MTRR A66G gene (*P* = 0.009) (Table 2). An association between A2756G MTR genotypes and parasitaemia was also found (*P* = 0.02). Individuals with 2756AA genotype predominantly (19/26) had high parasitaemia (>10,000 trophozoites/*μ*L of blood). The analysis of these polymorphisms according to the genus revealed a statistically significant difference for the MTHFR A1298C gene (*P* = 0.01) with more male subjects carrying the MTHFR 1298AA genotype (26/29 or 89.65%). Finally the data recorded in the present study were compared with previous data recorded in five other countries, namely, China, Pakistan, Brazil, South Korea, and Sweden (Table 3). The frequencies for MTHFR C677T recorded in the present study differed from those met in these previous studies. Hence the genotype CC was more encountered but the genotypes CT and TT were not frequently encountered. The frequencies for MTHFR A1298C are similar to those met in China and different from those of the other studies. The genotype MTR A2756G seemed to follow the same trends in both studies.

## 4. Discussion

This study described the MTHFR C677T, MTHFR A1298C, MTR A2756G, and MTRR A66G mutations. The genotypic frequencies found for the MTHFR C677T mutation are similar to those already reported in Burkina Faso [7]. Our results, however, differ from those reported in Sweden (46.3% 677CC, 43.4% 677CT, and 10.3% 677TT) in the mucosa of patients with colorectal cancer [13]; 28.6% 677CC, 44.9% 677CT, and 26.5% 677TT reported in patients with lymphoblastic leukemia in the Chinese population [17]; 51.4% 677CT, 41.4% 677CT, and 7.1% 677TT reported in Brazil in patients with chronic hepatitis C [14]; and 7.8% 677TT reported in children in the USA [18]. These differences could be explained by the type of population and the patients studied. Indeed, the minor allele “T” is more prevalent in the white and Hispanic population and almost absent in the black populations of Africa and the United States of America [19]. Frequencies of 88.1% and 11.9% for the A and C alleles were, respectively, observed for the MTHFR A1298C mutation in our study. These results are comparable to the allelic frequencies (87.4% A and 12.6% C) reported in 2002 by Simporè et al. [6] in Burkina Faso.

Genotypic frequencies for this gene (78.6% AA, 19% AC, and 2.4% CC) in our study are also similar to those found in 20–45 years of age (79.27% AA, 17.07% AC, and 3.66% CC) in Burkina Faso [7].

Our results, however, differ from the genotypic frequencies (66.8% AA, 29.3% AC, and 3.9% CC) reported in Chinese Han adults [11]; 67.8% AA, 30.3% AC, and 1.9% CC reported in Koreans with silent cerebral infarction [12]; and 20.8% AA, 48.7% AC, and 30.5% CC found in Pakistan [10]. These variations could be explained by ethnic origin and geographical areas.

The results of our study for the mutation MTR A2756G (61.9% AA, 33.3% AG, and 4.8% GG) are also different from the genotypic frequencies (52.4% AA, 38.8% AG, and 8.8% GG) reported in Pakistan [10] and in Jordan (83.50% AA, 14.46% AG, and 2.04% GG) [20] whereas they are similar to frequencies (63.6% AA, 31.3% GA, and 5.1% GG) reported in Sweden [13]. The genotypic frequencies of the MTRR A66G mutation revealed a 5% MTRR 66GG percentage close to the 6.6% reported in China [11] but different from the 34.5% reported in Sweden [13]. The small size of our study population and the uniqueness of our subjects (positive for malaria and not randomly selected in the general population) could explain these differences.

A correlation between MTRR A66G and hemoglobin genotypes was observed in this study. A2756G MTR genotype and parasitaemia showed a statistically significant association; MTR 2756AA individuals with predominantly high parasitaemia (>10,000 trophozoites/*μ*L of blood) could be more susceptible to malaria than others. An association between this mutation and malaria has been reported in the Northeast region of India [21]. These results demonstrate a probable selection of these polymorphisms under malaria pressure. A statistically significant difference in MTHFR A1298C genotypes by sex was also observed. Male individuals would be more affected by the MTHFR 1298AA genotype (26/29).

The small size of our study population because of limited financial resources therefore suggests the need for confirmation of these observations on a sufficiently large study population. The NFS results were comparable to those found in the general population. Only platelet and hemoglobin levels were used in this study to evaluate the correlations with the different mutations.

The effect of the mutations was studied on certain parameters such as variations in the number of white blood cells that may be due to other pathologies outside malaria which were difficult to assess. Despite the small sample size, this is the first time that all four polymorphisms have been studied in the same group of individuals in Ouagadougou, Burkina Faso.

Burkina Faso is a malaria endemic country with a high frequency of host resistance polymorphisms to* Plasmodium falciparum* malaria. Our study provided an overview of the genotypic frequencies of four major polymorphisms (MTHFR C677T, MTHFR A1298C, MTR A2756G, and MTRR A66G) for the genes involved in homocysteine metabolism correlated with simple malaria. Several associations such as MTRR A66G and hemoglobin genotypes; MTR A2756G and* Plasmodium falciparum*; and MTHFR A1298C infection and sex were observed and suggest a large enough sample for further analysis.

## Figures and Tables

**Table 1:** Sociodemographic and biological data.

Characteristics	*N*	(%)
Treatment		
Yes	22	52.38
No	20	47.62
Parasitaemia (parasite/*μ*L)		
<3,000	14	33.33
[3,000–10,000[	5	11.90
>10,000	23	54.76
Hemoglobin (g·L^−1^)		
<10	10	23.81
10–12	15	35.71
>12	17	40.48
Platelets count		
<15,000	14	33.33
≥15,000	28	66.67
Hemoglobin genotypes		
AA	24	57.14
AC	14	33.33
AS	2	4.76
CC	1	2.38
SC	1	2.38
Sex		
M	29	69.05
F	13	30.95
Age groups		
≤5	11	26.19
5–20	25	59.52
>20	6	14.28

**Table 2:** Correlation between genotypes and selected patient characteristics.

Genes	Genotypes	Hemoglobin genotypes					*P*	Parasitaemia			*P*	Sex		*P*
		AA	AC	AS	CC	SC		<3000	3000–10000	>10000		M	F	
MTHFR C677T	CC	20	12	0	2	2	*0.660*	16	6	14	*0.380*	22	14	*0.457*
	CT	4	0	0	0	0		0	0	4		4	0	
	TT	0	0	0	0	0		0	0	0		0	0	

MTHFR A1298C	AA	22	8	2	1	0	*0.160*	11	3	19	*0.605 *	26	7	***0.01***
	AC	2	5	0	0	1		3	2	3		2	6	
	CC	0	1	0	0	0		0	0	1		1	0	

MTR A2756G	AA	17	8	1	0	0	*0.717 *	4	3	19	***0.02***	19	7	*0.09*
	AG	6	5	1	1	1		9	2	3		10	4	
	GG	1	1	0	0	0		1	0	1		0	2	

MTRR A66G	AA	14	6	0	0	0	***0.009***	4	2	14	*0.185*	16	4	*0.182*
	AG	8	8	1	1	1		9	2	8		10	9	
	GG	0	0	1	0	0		1	0	0		1	0	

**Table 3:** Frequencies of mutations studied in different populations.

Country (reference)		Burkina Faso	Pakistan [10]	China [11]	South Korea [12]	Sweden [13]	Brazil [14]
Genes	Genotypes	%	%	%	%	%	%
MTHFR C677T	CC	**90.0**	71.3	32.8	28.5	55.9	53.8
	CT	**10.0**	26.2	43.9	52.9	35.8	40.4
	TT	**0.0**	2.5	23.3	18.6	8.4	5.7

MTHFR A1298C	AA	**78.6**	20.8	66.8	67.8	—	—
	AC	**19.0**	48.7	29.3	30.3	—	—
	CC	**2.4**	30.5	3.9	1.9	—	—

MTR A2756G	AA	**61.9**	52.4	—	—	64.8	—
	AG	**33.3**	38.8	—	—	30.9	—
	GG	**4.8**	8.8	—	—	4.3	—

MTRR A66G	AA	**50.0**	—	55.2	—	16.7	—
	AG	**45.0**	—	38.2	—	50.8	—
	GG	**5.0**	—	6.6	—	32.4	—
